# Opposition: Unfractionated heparin should no longer be used in the catheterization laboratory

**DOI:** 10.7603/s40602-014-0007-z

**Published:** 2014-05-24

**Authors:** Moo Hyun Kim, Young Seok Lee, Michael S. Lee

**Affiliations:** 1 Dept. of Cardiology, Dong-A University Hospital, Busan, Korea; 2 Departments of Pediatrics, Dong-A University Hospital, Busan, Korea; 3 UCLA Medical Center, Los Angeles, CA USA

## Abstract

The goal of anticoagulation during percutaneous coronary intervention (PCI) is the primary and secondary prevention of thrombotic and significant bleeding events that increase cardiovascular morbidity and mortality. Unfractionated heparin is the most commonly-used anticoagulant, but low-molecular weight heparin, and more recently bivalirudin are becoming increasingly popular in cardiac catheterization laboratories^1^.

The ACC/AHA/SCAI PCI guidelines^2^ recommend a 70-100 IU/kg bolus of heparin to achieve an activated clotting time (ACT) of 250-300 seconds for Hemotec and 300-350 seconds for Hemochron systems, when glycoprotein IIb/IIIa inhibitors are not used. When glycoprotein IIb/IIIa inhibitors are used, a bolus of 50-70 IU/kg of unfractionated heparin is recommended to achieve an ACT of 200-250 seconds.

## Case 1

A 65 year-old female was admitted to the emergency room due to severe resting chest pain. She had a past medical history of cervical cancer treated with radiotherapy, hypertension, diabetes and dyslipidemia. Her ECG showed Q waves in the inferior leads, mild ST segment elevation in lead III and aVR, and ST depression in V2-V5. Her CK-MB and troponin-I levels were elevated. The patient was in the OASIS-5 trial and was randomized to receive fondaparinux. Her angiogram revealed 3-vessel disease with a totally occluded distal right coronary artery, 75% stenosis in the mid left descending artery and 99% in the proximal circumflex artery, which was the culprit artery.

After predilatation with a 1.5 mm balloon, a large thrombus embolized to the left coronary system. The patient’s blood pressure dropped and cardiac resuscitation was initiated. The patient underwent emergent coronary artery bypass graft surgery.

## Alternative anticoagulation agents in the cardiac catheterization laboratory

Enoxaparin: The 2011 PCI guidelines^2^ give a class IIb recommendation when either subcutaneous administration is given upstream or IV administration is given at the time of PCI. There is also a class I recommendation for the use of additional IV administration in patients who are not fully anticoagulated at the time of PCI. In the SYNERGY trial^3^, enoxaparin was non-inferior to unfractionated heparin in reducing major adverse cardiac events with a modest increase in bleeding. The bleeding rate in the crossover group was higher than in the non-crossover group. Therefore, for upstream use of enoxaparin, the guideline recommends an additional 0.3 mg/kg IV bolus if the last dose was administered >8 h prior, and no additional dose if the last dose was administered <8 h prior.

Fondaparinux: In the OASIS-5 trial^4^, catheter thrombus formation during PCI was higherin the fondaparinux group than in the enoxaparin group (0.4% vs. 0.9%, p=0.001). Fondaparinux has a class III recommendation for PCI, but if used, additional antithrombotic therapy should be administered^2^.

Bivalirudin: In contrast to heparin, bivalirudin is a direct thrombin inhibitor which can neutralize clotbound thrombin and does not require a cofactor. The REPLACE-2 trial^5^ showed that bivalirudin, with provisional glycoprotein (GP) IIb/IIIa inhibitors, was non-inferior to heparin with GP IIb/IIIa inhibitor in reducing ischemic events, and caused less bleeding in patients undergoing elective PCI. In patients with ST-elevation MI undergoing primary PCI, bivalirudin alone when compared with heparin plus a GP IIb/IIIA inhibitor resulted in significantly reduced 30-day MACE and major bleeding events^6^. However, this drug is unavailable in many countries outside the US, including Korea, Japan and Singapore.

## Patients with renal impairment

Unlike unfractionated heparin which requires no dose adjustment for renal insufficiency, the maintenance dose of enoxaparin must be reduced to 1 mg/kg/day if the Creatinine Clearance (CrCl) <30 ml/min (Table. 1). The dose of bivalirudin may also need to be adjusted in patients with renal insufficiency.

## Case 2

A 75 year-old male was admitted to the hospital due to exertional chest pain. He had a long history of hypertension, and a history of pancreatitis and cholecystitis. He suffered a non-ST elevation myocardial infarction 2 months prior and had stent insertion at the proximal and mid anterior descending artery prior to admission. His coronary angiogram showed total occlusion at the distal right coronary with TIMI grade 2 collateral flow from the left anterior descending coronary artery (Fig. 1. upper left). Intervention was attempted via both femoral punctures. An antegrade approach attempt using a microcatheter and several guidewires was unsuccessful. Therefore the retrograde approach was attempted using the Finecross microcatheter (Terumo co., Japan) and a dedicated hydrophilic guidewire using the septal channel. A retrograde guidewire was advanced into an antegrade 7 Fr AR II guiding catheter after reverse controlled antegrade and retrograde subintimal tracking (CART) technique (Fig. 1. upper right, open arrow). Using the Rendezvous technique^7^, the antegrade guidewire was inserted into the distal right coronary total occlusion sites (Fig. 1. left lower). Successful PCI was performed with 3 drug-eluting stents (Fig. 1 right lower). During the retrograde technique, the ACT was measured every 30-60 minutes to achieve a target ACT >300 sec in order to prevent thrombotic complications^8^.

## Very high risk patients or those undergoing a prolonged complex procedure

For very high-risk patients, such as those requiring a left main coronary intervention or CTO retrograde intervention, unfractionated heparin might be the preferred antithrombin. In situations when the antegrade flow is compromised, bivalirudin might create a prothrombotic milieu in these coronary segments. This can lead to extensive intracoronary thrombus formation in areas of slow flow due to coronary dissection and prolonged dwell time with intracoronary hardware (wires, balloons, and stents)^9^. In addition, during retrograde CTO procedures, the prolonged presence of retrograde intracoronary hardware (Fig. 1, arrows) is prone to thrombus formation, possibly resulting in catastrophic thrombosis in the donor artery. In these situations, ACT-guided PCI using unfractionated heparin could be a safer strategy.

## Other issues

**Stent thrombosis:** Both ACUITY^10^ and HORIZON-AMI^6^ trials reported a significantly higher incidence of acute stent thrombosis in patients who received bivalirudin in comparison to UFH and GP IIb/IIIa inhibitors. In addition, data from HORIZON-SWITCH^11^ and the SCAAR registry^12^ suggest that administration of UFH together with bivalirudin may be required to reduce acute stent thrombosis^13^.

**Cost and antidote:** Bivalirudin is approximately 400 times more expensive than unfractionated heparin. The potential cost savings for each PCI is 500-1,000 euros^14^. In the event of acute bleeding, there is no agent for immediate reversal.

## Conclusion

Recent analysis from the SCAAR registry reported a higher adjusted odds ratio for 30-day mortality in bivalirudin compared to unfractionated heparin groups^14^. An old Korean saying states that “an old officer is a good officer”. Due to the very low cost and large amount of data, heparin is still a viable treatment option for PCI.

**Table. 1:** 2011 ACCF/AHA/SCAI PCI Guideline^2^

Antithrombin agents	Normal renal function	Dose adjustment in impaired renal function
Heparin	70 to 100 units/kg IV bolus, titrate to ACT 250 to 300 sec (50 to 70 units/kg IV if GP IIb/IIIa used, titrate to ACT 200 to 250 sec).	Renal adjustment: none.
Bivalirudin	0.75mg/kg IV bolus, then 1.75 mg/ kg/hr IV.	Renal adjustment of continuous infusion: CrCl 10 to 29 mL/min: 1mg/kg/hr; Hemodialysis dependent: 0.25mg/kg/hr.
Enoxaparin	Not generally started for elective PCI. For the occasional patient already on the drug, it can be continued at the prior dose with an additional 0.3 mg/kg IV if 8 to 12 hours since last dose.	Renal adjustment: avoid use if CrCl<30 mL/min or dialysis dependent

**Legend for Fig. 1.**
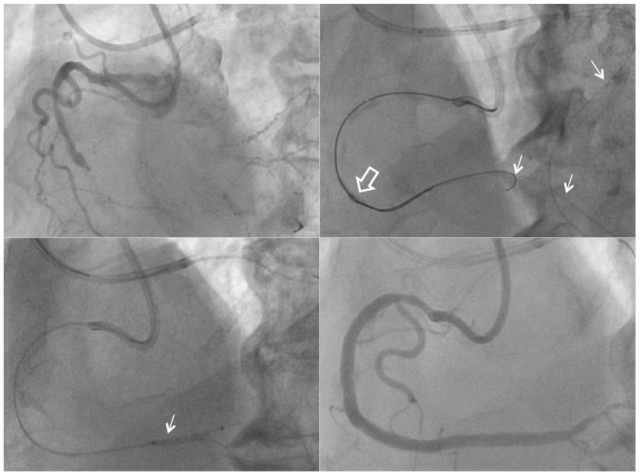
Coronary intervention of retrograde approach in a 75 year old male patient. Distal right coronary artery was totally occluded (left upper) and was completed with 3 stents (right lower). During the procedure reverse CART (right upper, open arrow) and Rendevous procedures were performed. Prolonged dwell time with intracoronary hardware (wires, balloons, and stents, arrows) requires strict ACT-guided anticoagulation to prevent thrombotic events.
